# Needles in the Haystack: Identifying Individuals Present in Pooled Genomic Data

**DOI:** 10.1371/journal.pgen.1000668

**Published:** 2009-10-02

**Authors:** Rosemary Braun, William Rowe, Carl Schaefer, Jinghui Zhang, Kenneth Buetow

**Affiliations:** 1 Laboratory of Population Genetics, National Cancer Institute, National Institutes of Health, Bethesda, Maryland, United States of America; 2 Center for Biomedical Informatics and Information Technology, National Cancer Institute, National Institutes of Health, Bethesda, Maryland, United States of America; The University of Queensland, Australia

## Abstract

Recent publications have described and applied a novel metric that quantifies the genetic distance of an individual with respect to two population samples, and have suggested that the metric makes it possible to infer the presence of an individual of known genotype in a sample for which only the marginal allele frequencies are known. However, the assumptions, limitations, and utility of this metric remained incompletely characterized. Here we present empirical tests of the method using publicly accessible genotypes, as well as analytical investigations of the method's strengths and limitations. The results reveal that the null distribution is sensitive to the underlying assumptions, making it difficult to accurately calibrate thresholds for classifying an individual as a member of the population samples. As a result, the false-positive rates obtained in practice are considerably higher than previously believed. However, despite the metric's inadequacies for identifying the presence of an individual in a sample, our results suggest potential avenues for future research on tuning this method to problems of ancestry inference or disease prediction. By revealing both the strengths and limitations of the proposed method, we hope to elucidate situations in which this distance metric may be used in an appropriate manner. We also discuss the implications of our findings in forensics applications and in the protection of GWAS participant privacy.

## Author Summary

In this report, we evaluate a recently-published method for resolving whether individuals are present in a complex genomic DNA mixture. Based on the intuition that an individual will be genetically “closer” to a sample containing him than to a sample not, the method investigated here uses a distance metric to quantify the similarity of an individual relative to two population samples. Although initial applications of this approach showed a promising false-negative rate, the accuracy of the assumed null distribution (and hence the true false-positive rate) remained uninvestigated; here, we explore this question analytically and describe tests of this method to assess the likelihood that an individual who is not in the mixture is mistakenly classified as being a member. Our results show that the method has a high false-positive rate in practice due to its sensitivity to underlying assumptions, limiting its utility for inferring the presence of an individual in a population. By revealing both the strengths and limitations of the proposed method, we elucidate situations in which this distance metric may be used in an appropriate manner in forensics and medical privacy policy.

## Introduction

In the recently published article “Resolving Individuals Contributing Trace Amounts of DNA to Highly Complex Mixtures Using High-Density SNP Genotyping Microarrays” [1], the authors describe a method by which the presence of a individual with a known genotype may be inferred as being part of a mixture of genetic material for which marginal minor allele frequencies (MAFs), but not sample genotypes, are known.

The method [1] is motivated by the idea that the presence of a specific individual's genetic material will bias the MAFs of a sample of which they are part in a subtle but systematic manner, such that when considering multiple loci, the bias introduced by a specific individual can be detected even when his DNA comprises only a small fraction of the mixture. More generally, it is well known that samples of a population will exhibit slightly different MAFs due to sampling variance following a binomial distribution; the genotype of the individual in question contributes to this variation, and so may be “closer” to a sample containing him than to a sample which does not. Based on this intuition, the article [1] defines a genetic distance statistic to measure the distance of an individual relative to two samples, summarized as follows:

Consider an underlying population 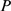 from which two samples 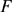 (of size 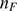) and 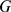 (of size 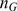) are drawn independently and identically distributed (i.i.d.) [in [1], these are referred to as “reference” and “mixture” respectively]. Consider now an additional sample 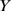; we wish to detect whether 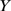 was drawn from 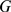, versus the null hypothesis that 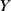 was drawn from 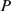 independent of 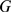 and 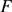. Given the MAFs 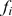 and 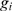 at locus 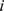 for 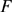 and 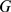, respectively, and given the MAFs 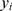 for sample 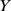 with 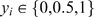 (corresponding to homozygous major, heterozygous, and homozygous minor alleles) at each locus 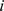, [1] defines the relative distance of sample 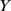 from 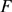 and 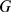 at 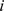 as:
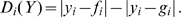
(1)By assuming only independent loci are chosen and invoking the central limit theorem for the large number of loci genotyped in modern studies, the article [1] asserts that the *z*-score of 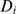 across all loci will be normally distributed,
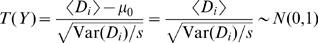
(2)where 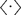 denotes the average over all SNPs 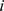, 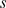 is the number of SNPs, and Equation 2 exploits the assumption [1] that an individual who is in neither 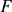 nor 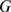 will be on average equidistant to both under the null hypothesis, i.e., 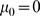. Per Equation 2, the null hypothesis that 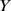 is in neither 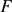 nor 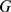 is rejected for values of 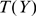 which exceed the quantiles of 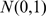 at the chosen significance level.

The article [1] proposes using this approach in a forensics context, in which 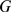 is a mixture of genetic material of unknown composition (e.g., from a crime scene), and 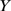 is suspect's genotype; by choosing an appropriate reference sample for group 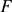, it is hypothesized that large, positive 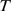 will be obtained for individuals whose genotypes are included in 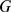, and hence bias 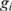, while individuals whose genotypes are not in 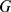 should have insignificant 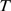 since they should intuitively be no more similar to the mixture sample 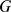 than they are to the reference sample 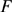. In [1], the authors applied this test to a multitude of individuals 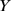, each of which are present in the samples constructed by them for 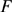 or 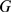, and report near-zero false negative rates. The article concludes that it is possible to identify the presence of DNA of specific individuals within a series of highly complex genomic mixtures, and that these “findings show a clear path for identifying whether specific individuals are within a study based on summary-level statistics.” In response, many GWAS data sources have retracted the publicly available frequency data pending further study of this method due to the concern that the privacy of study participants can be compromised. However, because no samples absent from both 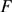 and 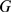 were used, false positive rates—significant 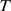 for individuals neither in 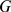 nor 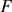
—are not assessed in practice; rather, they are simply assumed (Equation 2) to follow the nominal false-positive rate 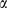 given by quantiles of the standard normal.

The conclusion that 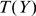 is comparable to a standard normal rests on several assumptions:

1. that 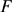, 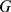 and 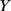 are all samples of the same underlying population 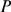;
2. that 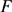 and 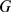 are similarly sized samples; and
3. that the SNPs 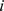 used to compute 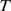 are independent.

Because these assumptions are difficult to control in practice, the effect of deviations from these assumptions is of interest. In this manuscript, we expand on [1] by investigating these effects both analytically and by applying Equations 1, 2 to null samples (those present in neither 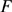 nor 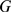). We also consider the accuracy of the classification when a relative of 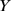 is present in sample 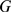.

Our tests reveal a good separation of the distributions for positive (i.e., in 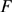 or 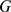) and null (in neither) samples, suggesting that a suprising amount of information remains in pooled data. However, our results indicate that membership classification via Equation 2 is sensitive to the underlying assumptions such that the distribution for null samples does not follow 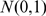, yielding misleadingly large 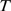 for null samples. As a result, applying the method from [1] is tricky in practice since additional information is often necessary to set appropriate thresholds for significance. Finally, we conclude with a discussion of the implications of our findings, both in forensics as well as regarding identification of individuals contributing DNA in GWAS.

## Methods

We explore the performance of the method described in [1] both analytically and empirically. For the empirical studies, we attempt to classify sample genotypes derived from publicly available data sources in order to assess the chances that an individual is mistakenly classified into a group which does not contain his specific genotype.

### Genotype data

2287 genotypes were obtained from the Cancer Genomic Markers of Susceptibility (CGEMS) breast cancer study. The samples were sourced as described in [2]. Briefly, the samples comprised 1145 breast cancer cases and a comparable number (1142) of matched controls from the participants of the Nurses Health Study. All the participants were American women of European descent. The samples were genotyped against the Illumina 550K arrays, which assays over 550,000 SNPs across the genome. To assess the genetic identity shared between samples, we computed the fraction of SNPs with identical alleles for all possible pairs of individuals; none exceeded 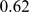.

Additionally, 90 genotypes of American individuals of European descent (CEPH) and 90 genotypes of Yoruban individuals were obtained from the HapMap Project [3]. In both cases, the 90 individuals were members of 30 family trios comprising two unrelated parents and their offspring. SNPs in common with those assayed by the CGEMS study and located on chromosomes 1–22 were kept in the analysis (sex chromosomes were excluded since the CGEMS participants were uniformly female); a total of 481,482 SNPs met these criteria.

### Classification of genotypes

The method as described in [1] and summarized in the Introduction was implemented using R [4]. Subsets of the data described above were used to construct pools 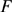 and 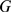, using the remaining genotypes as test samples for which the null hypothesis is true. A summary of the tests is provided in Table 1. In each test, SNPs which did not achieve a minor allele frequency 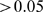 in both 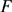 and 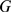 were excluded from the computation.

**Table 1:** Summary of tests performed.

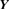 individuals	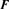 population	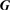 population	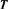 distribution
100 CGEMS cases not in 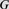	1042 CGEMS controls	1045 CGEMS cases	Figure 1
100 CGEMS controls not in 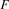			
90 HapMap CEPH			
90 HapMap YRI			
HapMap YRI mothers 16–30	HapMap YRI mothers 1–15 and fathers 1–15	HapMap YRI children 1–15 and fathers 16–30	Figure 2
HapMap YRI children 16–30			
HapMap CEPH mothers 16–30	HapMap CEPH mothers 1–15 and fathers 1–15	HapMap CEPH children 1–15 and fathers 16–30	Figure 2
HapMap CEPH children 16–30			

Summary of tests described. In the last four rows, the numbers refer to the families in the HapMap YRI and CEPH populations, such that child 1 is the offspring of mother 1 and father 1, et cetera.

## Results

The assertion that 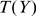 as given in Equation 2 follows a standard normal distribution under the null hypothesis that 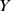 is in neither 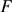 nor 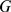 is based upon the assumptions that

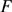
, 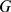 and 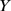 are all samples of the same underlying population 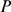;

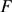
 and 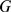 are similarly sized samples; and
3. the SNPs 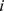 used to compute 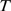 are independent.

We investigated the effect of deviation from these assumptions. A full treatment is presented in Text S1, and we summarize the results briefly here. In the case where 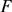, 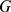, and 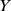 are not samples of the same underlying population, the differences in the minor allele frequencies of the source populations dominate 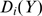 such that deviations from zero are no longer attributable to the subtle influence of *Y*'s presence in 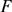 or 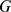. In the case where 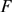, 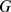, and 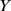 are samples of the same population but 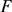 and 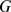 are of differing sizes, the larger one will be a more representative sample of the underlying population and hence closer, on average, to a future sample 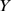. Both violations of assumptions 1 and 2 above will lead to non-zero 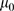 for null samples. Considering that the difference in 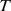 with and without the 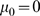 assumption in Equation 2 is
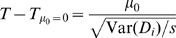
(3)and that the number of SNPs 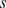 is on the order of 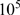, even slight deviations away from the assumed 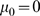 can have a pronounced effect when comparing 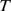 against a standard normal as given by Equation 2. Equation 2 also presumes that the SNPs are independent, such that the variance of the mean of 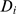 can be estimated as 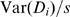 in the denominator of Equation 2; as shown in Text S1, even a slight average correlation amongst the SNPs (due, for instance, to linkage disequilibrium) will cause the distribution of 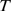 in practice to be much wider than that assumed in Equation 2, once again owing to the large number of SNPs considered. Because it appears that slight deviations from the assumptions outlined above may have a strong effect on the obtained 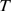 values, the false-positive rate of the method proposed in [1] may in practice be considerably higher than the nominal false-positive rate 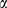 given by quantiles of 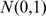.

### Empirical tests

To explore the performance of the method in realistic situations, we carried out the computations described by Equations 1,2 for various 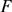, 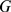, and 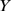 as described in Table 1. Distributions of 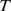 for each of the tests described in Table 1 are shown in the corresponding figures listed in the table. We find that while the distributions of in-*F*, in-*G* and in-neither values of 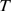 are distinct, calibrating thresholds for classifying an unknown sample is difficult without additional information. This is due to the fact that the distribution of 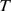 for null samples deviates strongly from a standard normal in practice.

We begin first by considering a best-case situation in which 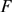 and 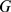 are both large samples of the same underlying population 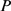, and the samples to be classified are also from 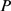. Here, we randomly select 100 cases and 100 controls from CGEMS to form an out-of-pool test sample set comprising 200 individuals, using the remaining 1045 CGEMS cases and 1042 CGEMS controls as pools 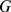 and 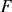, respectively. (Several such random subsets were created; the results were consistent and hence we present a single representative one.) 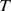 (Equation 1, 2) was computed for all the samples and compared to a standard normal (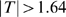 yields a nominal 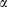 (*p*-value) of 0.05 and 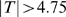 yields a nominal 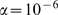). The sensitivity and specificities obtained are given in Table 2.

**Table 2:** Empirical sensitivity and specificity for the tests shown in Figure 1 assuming 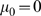.

	481,382 SNPs		50,000 SNPs	
	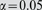	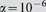	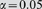	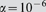
Sensitivity	99.8%	97.5%	96.3%	36.3%
Specificity, 200 CGEMS	31.0%	70.5%	79.0%	99.5%
Specificity, 90 HapMap CEPH	5.5%	27.7%	45.5%	100.0%
Specificity, 90 HapMap YRI	0.0%	0.0%	4.4%	97.7%

Classification results are given for two different nominal false positive rates 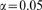 and 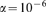.

**Figure 1:**
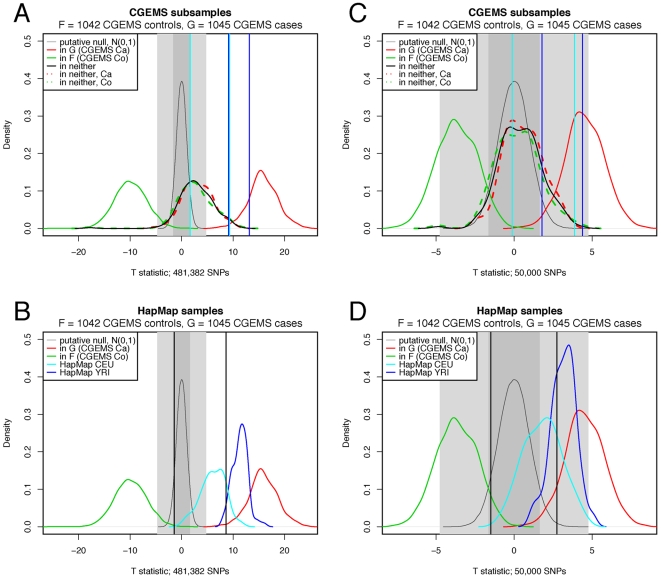
Comparison of *T* distributions. Comparison of *T* distributions for true positive and null samples versus putative null distribution, starting with 481,382 SNPs in (A,B) and 50,000 SNPs in (C,D). In all plots, true positive 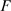 (1042 CGEMS controls) is shown as a solid green curve, true positive 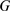 (1045 CGEMS cases) is shown as a solid red curve, and the putative null 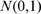 is given as a thin grey curve. The dark and light grey regions represent the areas for which the null hypothesis would be accepted at 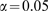 and 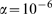, respectively. In plots (A,C), CGEMS test samples in neither 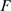 nor 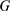 (100 CGEMS cases and 100 CGEMS controls) are given by a heavy black curve. The CGEMS case and CGEMS control distributions within this group are shown as dashed red and green lines, respectively. In plots (B,D), 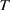 distributions are given for HapMap CEPHs (cyan) and YRIs (blue). Vertical lines mark the 0.05 and 0.95 quantiles of the negative CGEMS samples (black), HapMap CEPHs (cyan), and HapMap YRIs (blue).

Distributions of 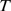 values for all three groups of CGEMS samples are shown in Figure 1A. Notably, the distributions of in-*F*, in-*G*, and in-neither samples are all quite distinct. For the positive samples (those in 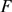 or 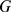), the classifier performs fairly well, correctly classifying 2083 samples (and calling 4 as in neither 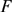 nor 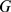). However, of the 200 test samples which were in neither 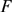 nor 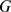, only 62 have 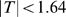; the rate of false positives is thus 69% if 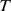 is used as an indicator of group membership under the assumptions in [1] at the nominal 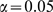 (see Table 2).

Next, we consider a less ideal, yet probable, case in which the null samples are not from the same underlying population 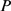. Here, we leave 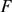 and 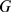 as above, and apply Equation 1, 2 to 90 HapMap American individuals of European descent (whom, one might assume, would be relatively similar to the Americans of European descent comprising groups 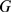 and 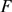). A plot of the 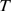 value distributions is given in cyan in Figure 1B. Again, there is little overlap with the true positive distributions, but when comparing the 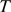 values against 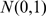, the sensitivity is quite low (see Table 2). A yet more extreme case, in which 90 HapMap Yoruban individuals were classified with respect to 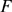 and 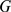, results in a distribution of 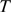 values that overlaps with the 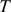 values from group 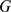 (Figure 1B, blue curve) and exceedingly low specificity (Table 2). We thus see in practice a strong dependence of 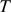 upon the assumption that 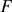, 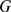, and 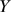 are samples of the same population.

The reason for the high false-positive rates in practice despite the stringent nominal false positive rate is clear from the plots Figure 1A and 1B: namely, it can be seen that the putative null distribution (light grey line, 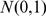, cf Equation 2) does not correspond to the observed distribution for samples for which the null hypothesis is correct, with differences in both the location and width.

The overall shift in the location of the distributions is a result of violations of the assumption that each sample 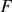, 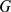, and 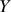 are drawn on from the same underlying population 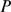. The magnitude of this effect is derived in Text S1 as 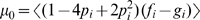, where 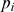 are the MAFs of the population from which 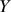 is drawn (hence the different rightward shifts of the CGEMS, CEPH, and YRI distributions). Because of the large number of SNPs 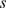 in Equation 2, small deviations from 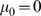 are magnified; even ancestrally similar populations, such as the 200 CGEMS test samples and the HapMap CEPHS, have different distributions of 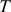.

The broadening of the 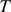 distribution is a result of correlation between SNPs. In Equation 2, it is assumed that the variance of the mean of 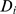 be estimable by the mean of the variance, ie, 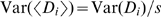, which is true for independent SNPs. However, if there exists average correlation 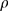 amongst the SNPs (due to linkage disequilibrium),
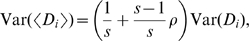
(4)which can be quite large even for small average correlation 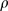 due to the high number 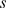 of SNPs. The result of increased LD is a broader distribution of 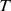 values, as observed in Figure 1A and 1B: we observe a narrower distribution of 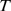 for the HapMap YRI samples versus the Caucasian CGEMS participants and HapMap CEPHs (the Yoruban individuals, who come from an older population, have lower average LD).

The effect of LD on the distribution of 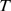 may be countered by selecting fewer SNPs; the results of this approach can be seen in Figure 1C and 1D and in Table 2. Here, 50,000 SNPs were selected, uniformly distributed across of the 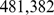 SNPs used in Figure 1A and 1B. 50,000 SNPs was shown in [1] to be a reasonable lower bound to detect at nominal 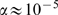 one individual amongst 1000, which is the concentration of true positive individuals in this test. As is clear from Figure 1, reducing the number of SNPs narrows the distributions considerably, yet at the same time brings them closer together such that the crisp separation previously obtained is reduced. Using this method, we see that the 200 CGEMS samples now have a distribution closer to that of the putative null 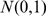 such that using a threshold of 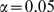 yields an improved—yet still larger than nominal—21% false-positive rate while maintaining a high 96.3% true positive rate. However, the misclassification rate is still over 50% for both HapMap samples, and improving these values requires compromising the sensitivity, a direct result of the overlapping 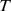 distributions for the 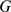 and HapMap samples.

Despite the low sensitivities obtained in our tests, it is apparent from Figure 1 that the true positive individuals have a significantly different distribution of 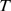 values than do the null samples, such that if appropriate thresholds were selected the classification could be improved (note that in practice, the distributions of the true positive individuals are unknown, since reconstructing them requires full genotypes, not just the MAFs, of 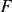 and 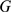). One simple apprach, motivated by the observed separation of distributions in Figure 1, would be to collect a set of presumed-null genotypes from which to estimate the null 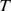 distribution. Consider a situation in which we have 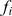 and 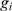, along with an individual 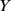 who is one of the 200 CGEMS samples not in 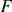 or 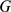, but no other genotypes. We might reasonably turn to publicly available HapMap genotypes as a group from which to construct an empirical null distribution for setting thresholds. The lines in Figure 1A and 1C depict this case. Using the 0.05 and 0.95 quantiles obtained from the HapMap CEPH 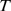 distribution (cyan bars) as thresholds improves the accuracy relative to using 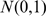 quantiles, but still incorrectly classifies half of the 200 CGEMS samples; the false positive rate is yet greater (and the true-positive rate smaller) when using the HapMap YRI quantiles (blue bars). Likewise, roughly a quarter of the HapMap CEPHs and the majority of HapMap YRIs lie outside the thresholds set from the 200 CGEMS samples in Figure 1B and 1D.

These examples, as well as the analytical results described in Text S1, show that deviations from the assumptions that 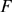, 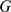, and 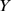 are i.i.d. samples of the same population 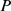 can produce misleadingly large values of 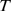. While Equations 1, 2 produce good separation of the 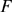, 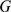 and null sample distributions, appropriately calibrating the thresholds for classification is difficult in practice.

#### Classification of relatives

**Figure 2:**
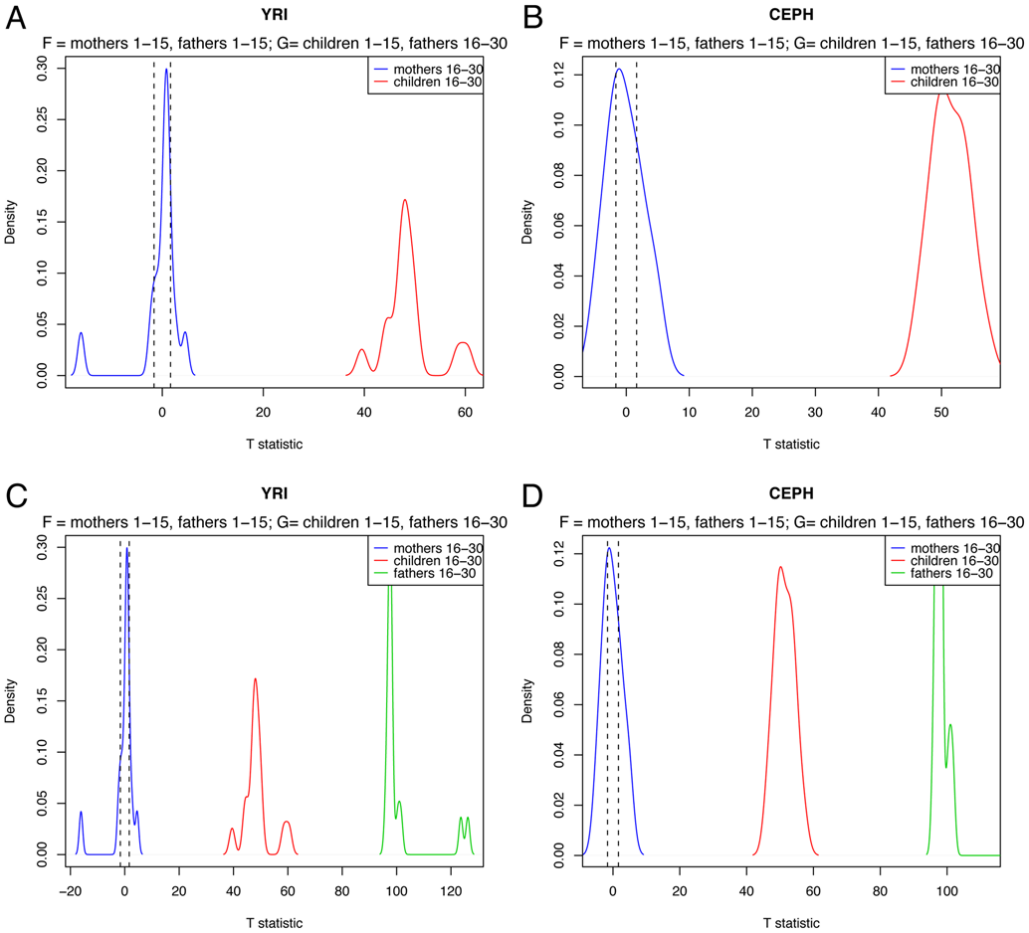
Distribution of *T*. Distributions of *T* for out-of-group samples who are related (red line) and unrelated (blue line) to individuals in *G* for HapMap YRI (A) and HapMap CEPH (B) populations. (C) and (D) show the same distributions as (A) and (B) respectively, with the addition (green line) of individuals who are in *G* and unrelated to *F* (i.e., true positives). Dashed black lines indicate the *T* significance thresholds of ±1.64 at nominal 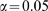.

We briefly consider the classification of individuals who are relatives of true positives. This can be investigated by using HapMap trios, since we can reasonably expect that the children will bear a greater resemblance to their parents than their parents do to one another. Recalling that the HapMap pools consist of thirty individual mother-father-offspring pedigrees, we construct pools as follows:

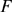
 = Mothers from pedigrees 1–15 and fathers from pedigrees 1–15

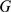
 = Children from pedigrees 1–15 and fathers from pedigrees 16–30

and then compute 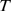 for mothers and children from pedigrees 16–30 using the same SNP criteria as before. The results of these tests for both the CEPH and YRI pedigrees, given in Figure 2, are as expected, with the children having a significantly higher distribution of 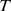 than the mothers; the 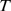 values for all the children were so large that *p*-values 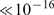 were obtained when comparing to 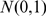. By contrast, 5/15 of the YRI mothers from pedigrees 16–30 and 10/15 of the CEPH mothers from pedigrees 16–30 yielded 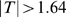 (with distributions roughly centered about 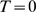). The wider distribution amongst the CEPHS again reflects the effect of LD. In Figure 2 we can see that the method has the power to resolve three groups: those in a group, those related to members of a group, and those who are neither. Note, however, that without having the distribution of 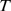 for true positives (which necessitates knowing the genotypes of true positives), it is not clear that setting a threshold to distinguish between true positives and their relatives is possible.

#### Positive predictive value of the method

**Figure 3:**
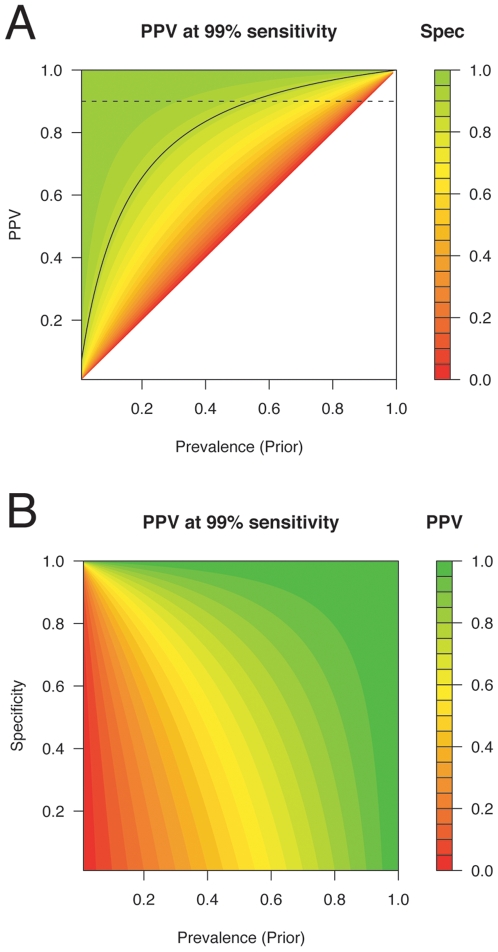
Positive predictive value (PPV) as a function of prevalence and specificity given 99% sensitivity. In (A), PPV is shown on the *y* axis and color corresponds to specificity. The black curve depicts the 87% sensitivity line—the best sensitivity obtained in the empirical tests in Table 2. In (B), PPV is shown by color, and the *y* axis corresponds to specificity.

The effect of the modest specificity—even in the best of cases described above—on the posterior probability that the individual 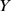 is in 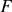 or 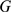 is considerable, given that the prior probability is likely to be relatively small in most applications of this method. Let us consider the positive predictive value (PPV), which quantifies the post-test probability that an individual 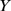 with a positive result (i.e., significant 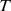) is in 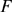 or 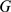. This probability depends on the prior probability that the individual is in 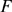 or 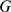, i.e., on the prevalence of being a member of 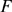 or 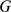. PPV follows directly from Bayes' theorem, and is defined as
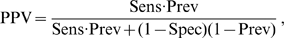
(5)where the PPV is the posterior probability that 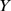 is in 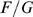 given a prior probability of 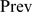. We can write this equivalently in terms of the positive likelihood ratio 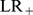,
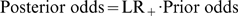
(6)

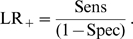
(7)A plot of PPV vs. prevalence is given in Figure 3. Even with the best sensitivity (96.3%) and specificity (79%) obtained in Table 2—that in which 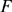, 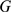, and 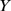 were strictly drawn on the same underlying population 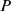, 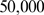 SNPs were used, and a nominal 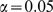 was used as a threshold—the prior probability (prevalence) of 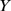 being in 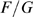 needs to exceed 66% in order to achieve a 90% post-test probability that the subject is in 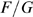. For a PPV of 99%, the prior probability needs to exceed 72% for any specificity under 95%, assuming the observed sensitivity of 99%. The low specificities obtained in practice thus require a strong prior belief that 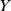 is in 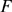 or 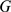.

The difference between the empirical false-positive rate and the nominal false-positive rate based on the standard normal has a strong effect on the posterior probabilities. Consider that 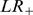 at 87% specificity and 99% sensitivity is 7.6, versus 990000 if the nominal false-positive rate 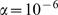 were correct. For prior probability of 1/1000, the first case yields a posterior probability of 1.1/1000, while the second yields a posterior probability of 998/1000. These differences, which are difficult to measure without additional, well-matched null sample genotypes and which depend strongly on the degree to which the assumptions underlying the method are met (consider the differences between the CGEMS and HapMap CEPH specificities in Table 2), pose a severe limitation on the utility of using Equations 1,2 to resolve *Y*'s membership in samples 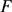 or 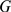.

## Discussion

In this work, we have further characterized and tested the genetic distance metric initially proposed in [1]. This metric, summarized here by Equations 1,2, quantifies the distance of an individual genotype 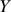 with respect to two samples 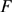 and 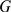 using the marginal minor allele frequencies 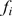 and 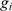 of the two samples and the genotype 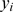. The article [1] proposes to use this metric to infer the presence of the individual in one of the two samples, and the authors demonstrate the utility of their classifier on known positive samples (i.e., samples which are in either 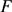 or 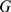) showing that in this situation their method yields classifications of high sensitivity. Our investigations confirm that the sensitivity is quite high (correctly classifying true positives into groups 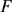 and 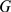) and that in-*F*, in-*G*, and null samples have distinct distributions of 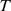 values. However, we also find that the distribution of 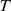 for null samples does not follow the presumed standard normal, and thus the specificity is considerably less than that predicted by the quantiles of the putative null distribution 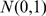. Calibrating a more accurate set of thresholds is difficult in practice, limiting the utility of Equations 1, 2 to positively identify *Y*'s presence in samples 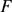 or 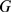.

In this work we have shown that high 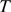 values, significant when compared against 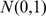, may be obtained for samples that are in neither of the pools due to violations of the assumptions that 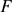, 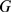 and 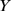 are all samples of the same underlying population; that 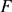 and 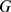 are similarly sized samples; and that the SNPs 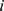 used to compute 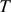 are independent. The high false positive rates in Table 2 result from deviations of the first and third assumptions. These assumptions are difficult to meet; for instance, HapMap CEPH and CGEMS samples are sufficiently dissimilar that the HapMap CEPH samples exhibit greater deviation from violations of the first assumption, despite the fact that both samples are Americans of European descent. Additionally, the conclusion that high 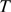 values result from *Y*'s presence in 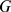 relies upon the questionable assumption that individuals in neither 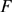 nor 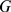 will be equidistant from both, resulting in false positives for relatives of true positive individuals, even when the other assumptions are met.

The low false positive rate in practice, resulting from the difficulty in accurately calibrating the significance of 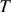, results in a likelihood ratio (and hence post-test probability) that is also low. When the prior probability of *Y*'s presence in 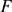 or 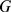 is modest, strong evidence (i.e., high specificity) is needed to outweigh this prior, which was not achieved in our tests. On the other hand, when samples were known *a priori* to be in one of the groups *F*/*G*, Equations 1,2 correctly identify the sample of which the individual is part.

These findings have implications both in forensics (for which the method [1] was proposed) and GWAS privacy (which has become a topic of considerable interest in light of [1]). We briefly consider each:

### Forensics implications

The stated purpose [1] of the method—namely, to positively identify the presence of a particular individual in a mixed pool of genetic data of unknown size and composition—is difficult to achieve. In this scenario, we have 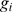 (from forensic evidence) and a suspect genotype 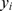. To apply the method, we would need 1) to assume that 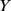 and 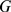 are indeed i.i.d. samples of the same population 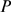; 2) to obtain a sample 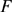 which is *also* a sample of the underlying population 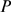, well-matched in size and composition to 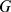; 3) to obtain an estimate of the sample size of 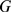 such that sample-size effects can be appropriately discounted (see Text S1); and 4) to assume that the *p*-values at the selected classification thresholds are accurate. The high false-positive rates which result from even small violations of these criteria make it exceedingly likely that an innocent party will be wrongly identified as suspicious; it is even more likely for a relative of an individual whose DNA is present in 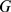.

### GWAS privacy implications

Here the scenario of concern is that of a malefactor with the genotype of one (or many) individuals, and access to the case and control MAFs from published studies; could the malefactor use this method to discern whether one of the genotypes in his possession belongs to a GWAS subject? In this case, 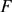 and 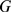 are known to be samples of the same underlying population 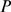 (due to the careful matching in GWAS), and their sample sizes are large and known. However, the malefactor still needs 1) to assure that 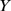 is a member of this population as well (as shown by the poor results when HapMap samples were classified using CGEMS MAFs) and 2) to assume that the *p*-values at the selected classification thresholds are accurate. Additionally, the prior probability that any of the genotypes in the malefactor's possession comes from a GWAS subject is likely to be quite small, since GWAS samples are a tiny fraction of the population from which they are drawn. Even if the malefactor were able to narrow down the prior probability to one in three, a sensitivity of 99% and a specificity of 95% is needed to obtain a 90% posterior probability that the individual is truly a participant.

On the other hand, if the malefactor *does* have prior knowledge that the individual 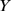 participated in a certain GWAS but does not know *Y*'s case status, Equations 1, 2 permit the malefactor to discover with high accuracy which group 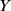 was in. Additionally, in the case of *a priori* knowledge, the participant's genotype is not strictly necessary, since a relative's DNA will yield a large 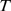 score that falls on the appropriate 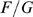 side of null.

Despite these limitations, we observe that the distributions of 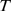 values for in-*F*, in-*G*, and null samples separate strongly, suggesting that each individual contributes a pattern of allele frequencies that remains in the pooled data. While calibrating thresholds to distinguish these distributions without additional information is not presently possible, the potential for more sophisticated methods to overcome these barriers cannot be discounted and presents an avenue for future work.

Moreover, we believe that the distance metric (Equations 1, 2) as presented may still have forensic and research utility. It is clear from both our studies and the original paper [1] that the sensitivity is quite high; in the (rare) case that a sample has an insignificant 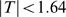, it is very likely that 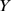 is in neither 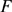 nor 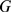. We can also see that genetically distinct groups have 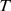 distributions with little overlap (Figure 1), and so it may be worth investigating the utility of Equations 1,2 for ancestry inference.

On this note, let us once more consider the quantity which Equation 1 measures, namely the distance of 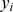 from 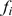 relative to the distance of 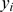 from 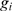. Referring to Figure 1A and 1C, we can see that samples 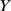 which are more like those in sample 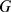 have a distribution that lies to the right of samples which are more similar to 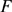, as expected; that is, in Figure 1A and 1C, the distribution of null (not in 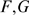) CGEMS cases (dashed red line) is shifted to the right with respect to the distribution of null CGEMS controls, as might be expected from Equation 1, i.e., the CGEMS case *Y*s are closer to CGEMS case *G*s than are the CGEMS control *Y*s. Although this difference is not statistically significant, one could imagine that it may be possible to select SNPs for which the shift is significant, i.e., a selection of SNPs for which unknown cases are statistically more likely to be closer (via Equation 1) to the cases in 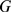 and unknown controls are statistically more likely to be closer to the controls in 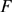. In this case, a subset of SNPs known to be associated with disease may potentially be used with Equations 1, 2 to predict the case status of new individuals; conversely, finding a subset of SNPs which produce significant separations of the test samples may be indicative of a group of SNPs which play a role in disease. Because this type of application would use fewer SNPs and would involve the comparison of two distributions of 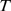 (cases 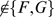 vs. controls 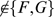), it may be possible to circumvent some of the problems stemming from the unknown width and location of the null distribution described above; still, much work is needed to investigate this possible application. If successful, the metric proposed in [1], while failing to function as a tool to positively identify the presence of a specific individual's DNA in a finite genetic sample, may if refined be a useful tool in the analysis of GWAS data.

## Supporting Information

Text S1 *D_i_* and *T* under the null hypothesis.
(0.22 MB PDF)
