# Supplementary material for: Needles in the Haystack: Identifying Individuals Present in Pooled Genomic Data
Source: PLoS Genet. 2009 Oct 2;5(10):e1000668. doi: 10.1371/journal.pgen.1000668 (PMC2747273; doi:10.1371/journal.pgen.1000668)
Supplement: Text S1 — Di and T under the null hypothesis. (0.22 MB PDF) [file pgen.1000668.s001.pdf]

Text S1: D_i and T under the null hypothesis

Here, we show analytically how deviations from the assumptions underlying Equations 1, 2 result in a null distribution which is not a standard normal.

Let us assume an underlying population P with MAFs p_i from which samples F (of size n_F) and G (of size n_G) are drawn i.i.d. Consider now an additional sample Y. The null hypothesis is that Y was drawn from P, independent of F and G; the alternative of interest is that Y is drawn from G (or, symmetrically, F). Under these idealized circumstances, we observe that:

$$f_i \sim \mathsf{Bin}(2n_F, p_i)/2n_F \,, \quad \text{(S1-1)}$$

$$g_i \sim \mathsf{Bin}(2n_G, p_i)/2n_G \,, \quad \text{(S1-2)}$$

$$y_i \sim \mathsf{Bin}(2, p_i)/2 \,, \quad \text{(S1-3)}$$

where the factors of two are a consequence of each sample possessing two independent alleles per locus. In [1], it is proposed that T (the Z-score of D_i across all SNPs) follows a standard normal distribution (Equations 1,2). This proposition rests upon two assumptions: namely, that the mean $\langle D_i \rangle$ across all SNPs under the null hypothesis is zero, i.e., $\mu_0 = 0$ in Equation 2; and that the SNPs i are completely independent such that we can write the variance of the mean as the mean variance, ie, $\mathsf{Var}(\langle D_i \rangle) = \mathsf{Var}(D_i)/s$ in the denominator of Equation 2. Below, we consider sources of deviation from $T \sim N(0, 1)$ under the null hypothesis.

Deviations from $\mu_0 = 0$

In the large-sample limit, under the null hypothesis,

$$\lim_{n_F \to \infty} f_i = p_i \; ; \; \lim_{n_G \to \infty} g_i = p_i \,, \quad \text{(S1-4)}$$

and hence

$$\lim_{n_F, n_G \to \infty} D_i = \lim_{n_F, n_G \to \infty} \big(|y_i - f_i| - |y_i - g_i| \big) = 0 \,. \quad \text{(S1-5)}$$

Intuition might further suggest that since f_i and g_i are both drawn from binomial distributions which are symmetric about p_i, any sampling deviations resulting from finite n_F, n_G will fall symmetrically, and hence $\mu_0 = 0$. As we will show below, however, this conclusion is sensitive to two assumptions:

1. that F, G and Y are drawn from the same underlying population;

2. that the sample sizes n_F and n_G are not only large, but comparable.

Because the number of SNPs s is quite large, slight deviations away from $\mu_0 = 0$ have the power to shift the location of the null distribution of T considerably, rendering T incomparable to a standard normal unless the true μ_0 is known. Consider that the difference in T with and without the $\mu_0 = 0$ assumption is

$$T - T_{\mu_0=0} = \frac{\mu_0}{\sqrt{\mathsf{Var}(D_i)/s}} \tag{S1-6}$$

and that because D_i ranges on $(-1, 1)$, $\max(\mathsf{Var}(D_i)) = 2$. This means that

$$\min(T - T_{\mu_0=0}) = \frac{\sqrt{s}}{\sqrt{2}}\mu_0 \tag{S1-7}$$

which can be quite large for even small values of μ_0 since the number of SNPs s is on the order of 10^5. It is thus essential that μ_0 be known or controllable.

Dependence of μ_0 on the assumption that F, G, and Y are samples of the same underlying population.

Let us write the difference between MAFs f_i and g_i at locus i as τ_i,

$$f_i = g_i + \tau_i\,. \tag{S1-8}$$

We can then write

$$D_i = |y_i - g_i - \tau_i| - |y_i - g_i|\ , \tag{S1-9}$$

and thus

$$\mu_0 = \langle |y_i - g_i - \tau_i| - |y_i - g_i| \rangle\,, \tag{S1-10}$$

where μ_0 is $\langle D_i \rangle$ under the null hypothesis.

We next make a simplifying assumption: since p_i are the *minor* allele frequencies and thus $0 \le p_i \le 0.5$, and since f_i and g_i are estimates of p_i, with few exceptions we will have $0 \le f_i \le 0.5$ and $0 \le g_i \le 0.5$ (eliminating this assumption does not significantly alter the results). Under this assumption we can write

$$|y_i - g_i - \tau_i| - |y_i - g_i| = \begin{cases} \tau_i & \text{for } y_i = 0; \\ -\tau_i & \text{for } y_i = 0.5; \\ -\tau_i & \text{for } y_i = 1. \end{cases} \tag{S1-11}$$

and hence Equation S1-10 may be written

$$\mu_0 = \sum_i \Big[\tau_i \cdot \mathbb{P}(y_i = 0|p_i) - \tau_i \cdot \mathbb{P}(y_i = 0.5|p_i) - \tau_i \cdot \mathbb{P}(y_i = 1|p_i)\Big]\, \mathbb{P}(p_i)\, \mathbb{P}(\tau_i)\,, \tag{S1-12}$$

where $\mathbb{P}(\cdot)$ denotes probability and where we have exploited the fact that because F, G are independent samples of P, τ_i is independent of p_i, i.e., $\mathbb{P}(\tau_i|p_i) = \mathbb{P}(\tau_i)$. Observing that

$$\begin{aligned}
&\mathbb{P}(y_i = 0|p_i) = (1 - p_i)^2\,;\\
&\mathbb{P}(y_i = 0.5|p_i) = 2p_i(1 - p_i)\,;\\
&\mathbb{P}(y_i = 1|p_i) = p_i^2\,,
\end{aligned} \tag{S1-13}$$

Equation S1-12 becomes

$$\mu_0 = \sum_i \left(1 - 4p_i + 2p_i^2\right) \tau_i \, \mathbb{P}(p_i)\, \mathbb{P}(\tau_i) \tag{S1-14}$$

$$= \langle (1 - 4p_i + 2p_i^2)\, \tau_i \rangle\,, \tag{S1-15}$$

which is readily verified by simulation.

Equation S1-15 implies that when τ_i deviates from zero, either due to systematic differences in F and G (i.e., violation of the assumption that both are drawn on the same population P) or due to sampling variation, the location of the null distribution of the test statistic given by Equation 2 will be shifted by an amount equal to $\langle (1 - 4p_i + 2p_i^2)\tau_i \rangle \cdot \sqrt{s/\mathsf{Var}(D_i)}$ relative to that under the assumption that $\mu_0 = 0$. It is important to note that the shift is a weighted average of τ_i; ie, it depends not only on the differences in MAFs τ_i but also on p_i, and hence it is not sufficient that $\langle \tau_i \rangle = 0$, since small τ_i will be amplified when p_i is small and reduced when p_i is large. As a result, predicting the deviation away from $\mu_0 = 0$ to properly calibrate T requires knowing not only $\tau_i = f_i - g_i$, but p_i as well.

In practice, τ_i is easily calculated. On the other hand, knowing p_i requires making assumptions about the population from which Y is drawn. In the case where Y is, in fact, drawn on the same population as F and G (and their sample sizes are known), f_i and g_i may be used to estimate p_i. However, when Y is from a different underlying population than are F and G, the p_i are difficult to obtain from the given data and the shift in T resulting from Equation S1-15 is not readily calculated.

As a demonstration of this correction, consider the results obtained under the $\mu_0 = 0$ assumption given in Table 2. If, instead, we recompute T using μ_0 as given by Equation S1-15 and assuming that $p_i = (n_F \cdot f_i + n_G \cdot g_i)/(n_F + n_G)$, the classification results become

	481,382 SNPs		50,000 SNPs	
	$\alpha = 0.05$	$\alpha = 10^{-6}$	$\alpha = 0.05$	$\alpha = 10^{-6}$
Sensitivity	99.90%	99.23%	97.36%	31.09%
Specificity, 200 CGEMS	40.0%	87.0%	78.0%	99.5%
Specificity, 90 HapMap CEPH	14.4%	55.5%	54.4%	100.0%
Specificity, 90 HapMap YRI	0.0%	0.0%	7.7%	100.0%

Dependence of μ_0 on sample sizes n_F and n_G.

The effect of deviations from the second assumption above is intuitively obvious: if $n_G > n_F$, G will better approximate the underlying population P and so will be closer on average to a future sample Y. The dependence is derived explicitly as follows:

Consider $\langle D_i \rangle$ (cf. Equation 1) under the null hypothesis assumptions that Y, F, and G are all drawn i.i.d. from the same underlying population P with MAFs p_i. Writing the probability distribution of p_i as $\mathbb{P}(p_i)$, $\langle D_i \rangle$ is given by

$$\langle D_i \rangle = \langle |y_i - f_i| - |y_i - g_i| \rangle = \langle |y_i - f_i| \rangle - \langle |y_i - g_i| \rangle \tag{S1-16}$$

$$\begin{aligned} &= \iiint_{-\infty}^{\infty} |y_i - f_i| \; \mathbb{P}(y_i|p_i)\, \mathbb{P}(f_i|p_i)\, \mathbb{P}(p_i)\, dy_i\, df_i\, dp_i - \\ &\qquad - \iiint_{-\infty}^{\infty} |y_i - g_i| \; \mathbb{P}(y_i|p_i)\, \mathbb{P}(g_i|p_i)\, \mathbb{P}(p_i)\, dy_i\, dg_i\, dp_i \;, \end{aligned} \tag{S1-17}$$

where we exploit the fact that Y, F and G are independent of each other but depend on the underlying population MAFs.

The dependence of the first (second) term in Equation S1-17 on n_F (n_G) is derived as follows. First, we note that since each y_i is two Bernoulli trials (two alleles) with probability p_i, we have the following values of $|y_i - f_i|$ with probability $\mathbb{P}(y_i|p_i)$ for each allowable value of y_i:

$$|y_i - f_i| \cdot \mathbb{P}(y_i|p_i) = \begin{cases} \big(1 - f_i\big) \cdot \big(p_i^2\big) & \text{for } y_i = 1\,; \\ \big(\,|0.5 - f_i|\,\big) \cdot \big(2p_i(1 - p_i)\big) & \text{for } y_i = 0.5\,; \\ \big(f_i\big) \cdot \big((1 - p_i)^2\big) & \text{for } y_i = 0\,. \end{cases} \tag{S1-18}$$

Moreover, since each f_i follows a binomial distribution of size $2n_F$ (two alleles per person), we invoke the normal approximation to the binomial for values of $n_F > 10$ with mean p_i and variance $p_i(1 - p_i)/(2n_F)$. Hence:

$$\mathbb{P}(f_i|p_i) = \sqrt{\frac{2n_F}{2\pi p_i(1 - p_i)}} \exp\left[-\frac{2n_F(f_i - p_i)^2}{2p_i(1 - p_i)}\right] \tag{S1-19}$$

$$= \frac{A_{F,i}}{\sqrt{\pi}} \exp\left[-A_{F,i}^2(f_i - p_i)^2\right] , \tag{S1-20}$$

where we introduce

$$A_{F,i} = \sqrt{n_F/(p_i(1 - p_i))} \tag{S1-21}$$

to simplify the notation. In consequence, the first term of Equation S1-17 can be written:

$$\iint_{-\infty}^{\infty} \left[(1-f_i)(p_i^2) + (|0.5-f_i|)(2p_i(1-p_i)) + (f_i)((1-p_i)^2)\right] \cdot \\ \cdot \frac{A_{F,i}}{\sqrt{\pi}} \exp\left[-A_{F,i}^2 (f_i - p_i)^2\right] \mathbb{P}(p_i)\, df_i\, dp_i \quad \text{(S1-22)}$$

and the second term may be written analogously for G. The absolute value in Equation S1-22 is dealt with by considering the $f_i \geq 0.5$ and $f_i \leq 0.5$ cases separately, i.e., treating Equation S1-22 as the sum of integrals

$$\int_{-\infty}^{\infty} \left[\int_{0.5}^{\infty} \Big((1-f_i)(p_i^2) + (f_i - 0.5)(2p_i(1-p_i)) + (f_i)((1-p_i)^2)\Big) \mathbb{P}(f_i|p_i)\, df_i + \\ + \int_{-\infty}^{0.5} \Big((1-f_i)(p_i^2) + (0.5 - f_i)(2p_i(1-p_i)) + (f_i)((1-p_i)^2)\Big) \mathbb{P}(f_i|p_i)\, df_i\right] \mathbb{P}(p_i)\, dp_i \quad \text{(S1-23)}$$

Expanding the polynomials in Equation S1-23 and once more using Equation S1-21 to simplify notation, we rewrite the above as

$$\int_{-\infty}^{\infty} \frac{A_{F,i}}{\sqrt{\pi}} \left[\int_{0.5}^{\infty} (C_1 f_i + C_2) e^{-A_{F,i}^2 (f_i - p_i)^2} df_i + \\ + \int_{-\infty}^{0.5} (C_3 f_i + C_4) e^{-A_{F,i}^2 (f_i - p_i)^2} df_i\right] \mathbb{P}(p_i)\; dp_i \quad \text{(S1-24)}$$

where C_1, C_2, C_3, and C_4 are functions of p_i but independent of f_i:

$$C_1 = 1 - 2p_i^2\,, \quad \text{(S1-25)}$$

$$C_2 = 2p_i^2 - p_i\,, \quad \text{(S1-26)}$$

$$C_3 = 1 - 4p_i + 2p_i^2\,, \quad \text{(S1-27)}$$

$$C_4 = p_i\,. \quad \text{(S1-28)}$$

Performing the interior integration in Equation S1-24 yields

$$\int_{-\infty}^{\infty} \frac{A_{F,i}}{\sqrt{\pi}} \left[(C_1 - C_3)\left(\frac{e^{-A_{F,i}^2 (0.5 - p_i)^2}}{2A_{F,i}^2}\right) + (C_3 p_i + C_4)\left(\frac{\sqrt{\pi}}{A_{F,i}}\right) + \\ + \Big((C_1 - C_3)p_i + (C_2 - C_4)\Big)\left(\frac{\sqrt{\pi}\,\mathrm{erfc}\big(A_{F,i}(0.5 - p_i)\big)}{2A_{F,i}}\right)\right] \mathbb{P}(p_i)\; dp_i\,. \quad \text{(S1-29)}$$

Expanding out the various Cs as well as $A_{F,i}$, we now have for the first term of $\langle D_i \rangle$

$$\int_{-\infty}^{\infty} (p_i(1-p_i)) \left[2\sqrt{\frac{p_i(1-p_i)}{\pi\, n_F}} \exp\left(-\frac{n_F(0.5-p_i)^2}{p_i(1-p_i)} \right) + \right. \\ \left. + 2(1-p_i) + (2p_i - 1)\mathrm{erfc}\left(\sqrt{\frac{n_F(0.5-p_i)^2}{p_i(1-p_i)}} \right) \right] \mathbb{P}(p_i)\, dp_i \,, \quad \text{(S1-30)}$$

which has an indirect dependence on n_F. Performing the same integration for the second term in Equation S1-17 yields analogous indirect n_G dependence. As a result, when $n_F < n_G$, the first term is greater than the second, yielding $\langle D_i \rangle > 0$; in the limit $n_F, n_G \to \infty$, this difference becomes smaller.

The dependence is illustrated by simulation in Figure S1-1A. Here, we assume a uniform distribution of p_i on $(0, 0.5)$ and construct 10^5 p_i's for the underlying population P from which we draw, independently, a sample G of size $n_G = 1000$ and 200 samples Y from which we estimate $\langle D_i \rangle$ under the null hypothesis. Sample F is drawn i.i.d. from P with sample sizes ranging from $n_F = 10$ to $n_F = 1000$, permitting us to plot $\langle D_i \rangle$ as n_F is varied. The simulation results are shown as circles, overlayed with a plot of Equation S1-17 using the result in Equation S1-30 and assuming the uniform distribution of p_i. The values for $\langle D_i \rangle$ obtained from the simulation closely matches those derived from Equation S1-30. In Figure S1-1B, the corresponding values of T are presented.

Deviations from $\mathsf{Var}(\langle D_i \rangle) = \mathsf{Var}(D_i)/s$

Invocation of the central limit theorem to compare T to a standard normal distribution (as given in Equation 2) requires that the variance of the mean of D_i be estimable by the mean of the variance, ie, $\mathsf{Var}(\langle D_i \rangle) = \mathsf{Var}(D_i)/s$. This, in turn, requires that the D_i are uncorrelated. However, if the various D_i are correlated—most notably due to linkage disequilibrium—this is no longer true. Specifically, the variance of the mean for s variables D_i with variance $\mathsf{Var}(D_i)$ and average correlation ρ amongst the distinct D_i is given by

$$\mathsf{Var}(\langle D_i \rangle) = \left(\frac{1}{s} + \frac{s-1}{s}\rho \right) \mathsf{Var}(D_i)\,. \quad \text{(S1-31)}$$

In the case where the average correlation amongst the D_i's is zero, Equation S1-31 yields the result which is found in the denominator of Equation 2; on the other hand, $\rho \neq 0$ generates a $\big(1 + (s-1)\rho\big)$ multiplicative increase over the correlationless variance. The large number of SNPs s results in little room for any correlation between them: consider that Equation S1-31 dictates that for a modest number of SNPs $s = 5 \cdot 10^4$ even a very slight

average correlation between all pairs of SNPs $\rho = 0.002$ would result in a tenfold increase in $\mathsf{Var}(T)$; for 500K SNPs ($s = 5 \cdot 10^5$), $\rho = 0.0002$ causes a a two order of magnitude increase in $\mathsf{Var}(T)$. However, it is impossible to ascertain ρ simply from y_i, f_i, and g_i. Instead, this issue may be addressed by choosing fewer SNPs and assuming that ρ is sufficiently small.

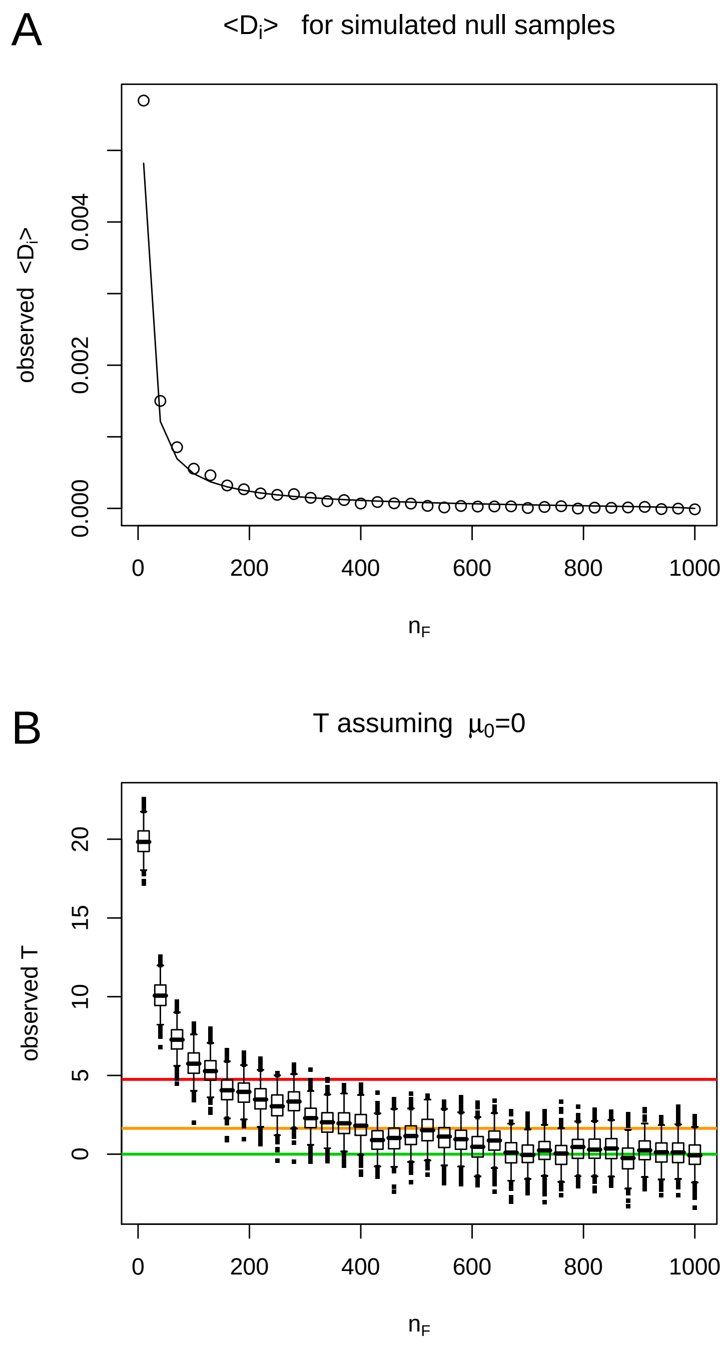

Figure S1-1: Observed $\langle D_i \rangle$ and T values for simulated data with varying sample sizes of n_F under the $\mu_0 = 0$ assumption. In A, open circles represent the average $\langle D_i \rangle$ for each simulation; the solid line is the theoretical $\langle D_i \rangle$ based on numerical integration of Eq. S1-30. In B, boxplots of the observed Ts for each simulation are given assuming $\mu_0 = 0$; box boundaries correspond to the 0.25 and 0.75 quantiles, and whiskers indicate the 0.05 and 0.95 quantiles (T values outside those limits are shown as square points). Horizontal lines at $T = 0$ (green), $T = 1.64$ (corresponding to $\alpha = 0.05$, in amber), and $T = 4.75$ (corresponding to $\alpha = 10^{-6}$, in red) are shown for reference; note that for $n_F < 600$, at least 25% of null samples yield significant T at the nominal $\alpha = 0.05$.
